# Knockdown of PPARδ Induces VEGFA-Mediated Angiogenesis *via* Interaction With ERO1A in Human Colorectal Cancer

**DOI:** 10.3389/fonc.2021.713892

**Published:** 2021-10-12

**Authors:** Wenjun Luo, Diao He, Jianhao Zhang, Zida Ma, Keling Chen, Zhaoying lv, Chuanwen Fan, Lie Yang, Yuan Li, Zongguang Zhou

**Affiliations:** ^1^ Department of Gastrointestinal Surgery, West China Hospital, Sichuan University, Chengdu, China; ^2^ Institute of Digestive Surgery, State Key Laboratory of Biotherapy and Cancer Center, West China Hospital, Sichuan University, Chengdu, China; ^3^ Laboratory of Liver Transplantation, Frontiers Science Center for Disease-Related Molecular Network, West China Hospital, Sichuan University, Chengdu, China

**Keywords:** peroxisome proliferator-activated receptor β/δ/D, angiogenesis, VEGFA, endoplasmic reticulum oxidoreductase 1 alpha, colorectal cancer

## Abstract

Angiogenesis is an important mechanism underlying the development and metastasis of colorectal cancer (CRC) and has emerged as a therapeutic target for metastatic CRC (mCRC). Our recent studies found that Peroxisome proliferator-activated receptor β/δ/D (PPARδ) regulates vascular endothelial growth factor A(VEGFA) secretion and the sensitivity to bevacizumab in CRC. However, its exact effect and underlying mechanisms remain unidentified. In this study, we showed that PPARδ expression was inversely associated with the microvascular density in human CRC tissues. Knockdown of PPARδ enhanced VEGFA expression in HCT116 cells and HUVEC angiogenesis *in vitro*; these phenomena were replicated in the experimental *in vivo* studies. By tandem mass tag (TMT)-labeling proteomics and chromatin immunoprecipitation sequencing (ChIP-seq) analyses, endoplasmic reticulum oxidoreductase 1 alpha (ERO1A) was screened and predicted as a target gene of PPARδ. This was verified by exploring the effect of coregulation of PPARδ and ERO1A on the VEGFA expression in HCT116 cells. The results revealed that PPARδ induced VEGFA by interacting with ERO1A. In conclusion, our results suggest that knockdown of PPARδ can promote CRC angiogenesis by upregulating VEGFA through ERO1A. This pathway may be a potential target for mCRC treatment.

## Introduction

Colorectal cancer (CRC) is the third most common malignant tumor and the second highest cause of cancer-associated death worldwide, with estimated more than 1.8 million new patients as well as over 800,000 deaths in 2018 (1). At initial diagnosis approximately 20-25% of patients had distant metastatic CRC (mCRC), and 50-60% of patients will eventually develop metachronous distant metastasis even after curative resection of the primary cancer (2, 3). Patients with mCRC without any treatment have a median survival time of 5-6 months (4). Targeting tumor angiogenesis has been shown to be an important strategy for mCRC treatment (5). Combining antiangiogenic drugs with established chemotherapeutic regimens has increased the median overall survival of mCRC patients from 12 months in the mid-1990s to almost 30 months (6, 7). Unfortunately, these antiangiogenic drugs fail to elicit long-lasting clinical responses in most patients due to primary or acquired resistance (8–10). The underlying mechanisms of therapeutic resistance remain unclear. However, this gap in knowledge is partly a result of the poor understanding of the molecular mechanisms of altered angiogenesis in the tumor microenvironment. Therefore, it is imperative to explore the complex angiogenic mechanisms of CRC to provide more potential targets for the development of therapeutic treatments.

Peroxisome proliferator-activated receptor β/δ/D (PPARδ), one of the nuclear hormone receptor superfamilymembers, is a ligand-dependent transcription factor. PPARδ plays a role in regulating fatty acid catabolism, energy homeostasis, cell differentiation, inflammation and tumorigenesis (11, 12). Previous studies have implicated PPARδ in the carcinogenesis of CRC; however, the results regarding the relationship of PPARδ with CRC are conflicting. Some studies support PPARδ as a promoter of CRC carcinogenesis, while other studies have reported the opposite results (13–22). In a series of studies, we found that the high PPARδ expression was linked to longer survival in patients with primary cancers, and PPARδ suppressed the proliferation and facilitated the differentiation of CRC cells (23–25). Our studies support the inhibitory role of PPARδ in CRC tumorigenesis.

Recently, we demonstrated a correlation between PPARδ and vascular endothelial growth factor A (VEGFA), which is recognized as a key contributor to the process of angiogenesis and a critical therapeutical target for mCRC (26). Our study revealed that knockdown of PPARδ promoted VEGFA secretion and reduced the sensitivity to bevacizumab (27). The low expression levels of PPARδ in vascular endothelial cells of CRC were associated with the increased expression of VEGFA (28). These studies indicate that PPARδ is involved in angiogenesis in CRC, but its exact role in CRC angiogenesis still need be defined.

In the present study, we found that the expression of PARRδ inversely correlated with CRC-associated angiogenesis. Furthermore, our results demonstrate that knockdown of PARRδ promoted CRC angiogenesis. Moreover, we combine proteomics with ChIP-Seq analyses to define a definitive molecular mechanism for PPARδ in regulating CRC angiogenesis. We identified that PPARδ suppress the expression of VEGFA mediated by endoplasmic reticulum oxidoreductase 1 alpha (ERO1A), an oxidase located in the endoplasmic reticulum. Our study suggests that PARRδ may be considered as a potential target for anti-angiogenic therapy in CRC.

## Materials and Methods

### Patients and Specimens

Human tissue specimens and patient information were obtained from the Department of Gastrointestinal Surgery, West China Hospital (Chengdu, Sichuan, China). A total of 120 patients with primary CRC were enrolled in this study between 2009 and 2011. No patients had received adjuvant treatment prior to surgery. All informed consent was acquired from patients, and Biomedical Ethics Committee of West China Hospital approved this study. Histological examination verified the diagnosis of CRC. Tumor staging were defined by two experienced pathologists independently based on the 8th edition of the AJCC (29, 30). All tissue samples were snap-frozen in liquid nitrogen and conserved at -80°C until further use. Detailed information on the patients and tumors is shown in the **Supplementary Table 1**.

### Immunohistochemical (IHC) Analysis and Microvessel Counting

Immunohistochemistry was conducted as described before (25). Briefly, slides were incubated with the diluted primary antibodies anti-PPARδ (SC-74517, CST) and anti-CD31 (ab76533, Abcam), followed by HRP-linked secondary antibody incubation. The IHC slides were examined by two independent pathologists according to our previous study (25). Each investigator assessed the proportion of cells stained and the intensity of staining in the whole section. The intensity in epithelial cells or tumor cells was scored as 0 (negative staining), 1 (weak staining exhibited as light yellow), 2 (moderate staining exhibited as yellow brown), and 3 (strong staining exhibited as brown).The proportion of cells stained was accessed using a 5 scoring system:0 (no positive cells), 1 (<10% positive cells), 2 (10%–40% positive cells), 3 (40%–70% positive cells), and 4 (>70% positive cells). The immunostaining intensity score and the percentage of positive cells were scored. The two scores were multiplied to obtain an immunostaining score that ranged from 0 to 12.

Microvessel density (MVD) was determined by Weidner’s methods (31). Briefly, the stained sections were first screened at low power (100× magnification) to determine the area of most intense staining of the tumor microvessel. Individual microvascular counts of the most intensely stained areas were obtained in a high-power magnification (200×) field (three fields per tumor section). All brown-stained endothelial cell and endothelial cell cluster, obviously separating from adjacent cells, were also calculated as a single, countable neovessel. The highest microvessels’ number was recorded as MVD in any 200× field. Each sample was separately examined, then scored by two pathologists. If there was a discrepancy in individual scores, a discussion was held to reach a consensus.

### Cell Lines and Culture

The human CRC cell lines (SW480, HCT116, SW620, HT29, and T84) used in our study were purchased from Procell Life Sciences Co. Ltd. (Wuhan, Hubei, China) and STR analysis was used to authenticate them by Procell Life Sciences Co. Ltd. (**Supplementary Material**). Dr. Lei Dai provided Human umbilical vein endothelial cells (HUVECs) (State Key Laboratory of Biotherapy, Sichuan University, Chengdu, Sichuan, China). The SW480, HCT116,SW620, HT29 and T84 cell lines were cultured in DMEM containing antibiotics and 10% fetal bovine serum (FBS). HUVECs were cultured in EndoGRO-VEGF medium (Millipore, MA, USA). All cell lines were incubated in a 37°C humidified incubator containing 5% CO2.

### Cell Transfection

The LV-PPARδ-shRNA-Puromycin (PPARδ-shRNA), LV-Flag-PPARδ-Puromycin (PPARδ), LV- ERO1A-shRNA-Puromycin (ERO1A-shRNA), LV-Flag- ERO1A-Puromycin (ERO1A) and negative control viruses were purchased from GeneChem Co., Ltd. (Shanghai, China). The shRNA targeting sequences were as below: PPARδ (#1) 5ʹ-GCTGGAGTACGAGAAGTGTGA-3ʹ,

PPARδ (#2) 5ʹ-GCATGTCACACAACGCTATCC-3ʹ,

PPARδ (#3) 5ʹ-GCTGGCCTCTATCGTCAACAA-3ʹ,

ERO1A (#1) 5ʹ-GGGCTTTATCCAAAGTGTTAC-3ʹ,

ERO1A (#2) 5ʹ-GCATTTGAGTGCAAGATATCT-3ʹ,

and ERO1A (#3) 5ʹ-GCCGTGTCCTTTCTGGAATGA-3ʹ. The lentiviruses were transfected into CRC cells and selected in medium with 2 µg/mL puromycin. The expression of PPARδ and ERO1A in transfected cells was validated by Western blot assays.

### Western Blot Analysis

Total cellular proteins were extracted using lysis buffer containing phenylmethylsulfonyl fluoride (PMSF) and phosphatase inhibitor. One hundred micrograms of protein were separated by SDS-PAGE and transferred onto PVDF membranes according to the method described previously. The blots were blocked for 1 h by 5% skimmed milk in TBST and incubated with various primary antibodies, including anti-PPARδ (SC-74517, CST) and anti-ERO1A (3264, CST), at 4°C overnight. Next, the blots were incubated for 1 h at room temperature with appropriate secondary antibodies (CST, MA, USA). The protein bands were visualized using a chemiluminescence kit (Beyotime Biotechnology, China).

### Preparation of Conditioned Medium (CM) and ELISA

The selected CRC-PPARδ-knockdown stable cells and CRC control cells were cultured for 72 h in 6-well plates. Thereafter, the supernatants were harvested and centrifuged at 3500 rpm for 5 min and then kept at -80°C until it was used as tumor cell-conditioned medium. VEGFA derived from tumor in the medium was measured by enzyme-linked immunosorbent assay (ELISA). The VEGFA concentration was measured with an ELISA Kit (Ab119566, Abcam, UK) according to the procedure described by the manufacturer.

### HUVEC Tube Formation Assay

Matrigel (ECM625, Merck Millipore, USA) was thawed on ice overnight, dispensed onto 96-well plates (50 μL/well) and polymerized at 37°C for 1 h. HUVECs (2×10^4^ cells/well) were seeded onto the Matrigel layer and cultured in HUVEC culture medium or tumor cell-conditioned medium that was collected earlier at a 1:1 ratio. Tube formation was observed after 7 hours’ incubation at 37°C, and imaging was performed by an inverted microscope (Olympus Corporation, Tokyo, Japan). The results, including the number of tube nodes (the intersection among 3 or more tubes) in a 100× field, were analyzed by using ImageJ software.

### Matrigel Plug Assay and Immunofluorescence (IF)

BALB/c nude mice (4 weeks old, Male) were purchased from the National Laboratory Animal Center (Beijing, China) and housed in SPF conditions. All animal care and handling procedures followed the National Institutes of Health Guide for the Care and Use of Laboratory Animals. All animal experiments were approved by the Animal Care and Use Committee of West China Hospital, Sichuan University. In brief, mice were subcutaneously injected with control and PPARδ-knockdown HCT116 cells (5×10^6^) resuspended in 500 µL of solution containing 80% growth factor-reduced Matrigel (356231, Corning, USA). Seven days later, mice were sacrificed, and Matrigel plugs were then dissected and stored at -80°C. Subsequently, plugs were embedded with optimal cutting temperature (OCT) compound (Miles, Elkhart, IN), cryostat sectioned, and incubated with anti-CD31 antibody. After that, the tissue sections were incubated in 5% BSA away from light with secondary fluorescent antibody (Invitrogen, 594 nm) at room temperature for 1 h. Finally, the nuclei were counterstained with DAPI (Thermo, MA, USA) for 30 min. Fluorescence microscope was used to visualized the fluorescence images (Olympus Corporation, Tokyo, Japan).

### *In Vivo* Tumor Xenograft Study

BALB/c nude mice (4 weeks of age, Male) were purchased from the National Laboratory Animal Center (Beijing, China). 3×10^6^ cells (HCT116 control cells and HCT116-PPARδ-knockdown cells) resuspended in 200 μL of PBS were injected into the right flanks of mice subcutaneously. Every 5 days, tumor volume was measured with a caliper and calculated with the following formula: Volume (mm^3^) = length × width^2^/2. 25 days after injection, all mice were sacrificed. Tumors were weighed and fixed with formalin. Tumor IHC staining and MVD were performed as described before.

### Protein Identification and Quantification by LC-MS/MS

Control or PPARδ-knockdown HCT116 cells were collected and sonicated on ice in lysis buffer (8 M urea, 1% protease inhibitor cocktail) with a high-intensity ultrasonic processor (Scientz). The supernatant was then harvested, and the concentration of protein was evaluated by a BCA kit following the instructions of manufacturer. After trypsin digestion and labelled by tandem mass tag/isobaric tag for relative and absolute quantitation (TMT/iTRAQ), the tryptic peptides were isolated with an EASY-nLC 1000 UPLC system. Then the peptides were subjected to NSI source followed by tandem mass spectrometry (MS/MS) in Q ExactiveTM Plus (Thermo Fisher Scientific, USA) coupled online to the UPLC. Next, the acquired MS/MS data were analyzed with the MaxQuant search engine (v.1.5.2.8). Finally, Tandem mass spectra were then searched against the human UniProt database which was concatenated with a reverse decoy database. The minimum score for modified peptides was set at > 40, and FDR was adjusted to < 1%.

### Bioinformatics Analysis of Differentially Expressed Proteins

A 1.2-fold increase or decrease in protein expression indicated significant differences between the groups. Gene Ontology (GO) enrichment and Kyoto Encyclopedia of Genes and Genomes (KEGG) pathway enrichment analyses were carried out on the basis of Fisher’s exact test after identifying corresponding gene symbols or gene IDs by InterProScan.

### ChIP-Seq Assay

DNA samples were collected from the control and PPARδ-knockdown HCT116 cells and prepared for Illumina sequencing on Illumina HiSeq 4000 following the protocol of HiSeq 3000/4000 SBS Kit (300 cycles). Then the sequencing images were produced by the sequencing platform. The image analysis and base calling were conducted with Off-Line Basecaller software (OLB V1.8). Statistical significance of ChIP-enriched regions were determined by comparisons of the IP with input samples or compared with a Poisson background model using a p-value threshold of 10^-4^.

### Statistical Analysis

Data are represented as the means ± SDs. Statistical analysis and graphs were performed with SPSS 20.0 and GraphPad Prism 7 software. Comparisons between the two groups was analyzed by Student’s t-test. All experiments were performed in triplicate. P-values less than 0.05 were considered to be significant difference.

## Results

### PPARδ Expression Is Inversely Correlated With Angiogenesis in Human CRC Tissues

Previously, PPARδ was detected mainly in the cytoplasm of epithelial cells, and its expression was increased in cancer tissues compared to normal mucosa (25). To explore the effect of PPARδ on angiogenesis, the expression of PPARδ and CD31, a surface marker of neovascular endothelial cells, was examined by IHC staining of 120 CRC tissues. Then, MVD was calculated. As a survival analysis was included in our previous study, we did not perform a survival analysis in this study (25).

**Figure 1:**
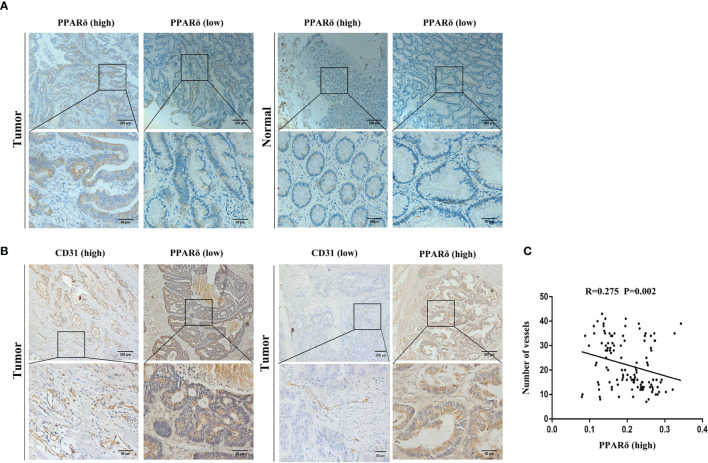
PPARδ expression is inversely correlated with angiogenesis in human CRC tissues. **(A)** Immunohistochemical staining of PPARδ protein in tumor tissues and paired normal tissues were evaluated (scale: 200 μm). **(B)** PPARδ expression level and MVD (CD31-positive cells) in tumor tissues (scale: 200 μm). **(C)** The correlation between the expression of PPARδ and MVD in 120 CRC patients was analyzed (P = 0.0002). Data represent the mean ± SD.

By IHC, we found that high expression of PPARδ was associated with early-stage disease, while low PPARδ expression was related to advanced-stage disease (**Figure 1A**). This result was consistent with our previous study (24). Moreover, we found increased MVD in CRC tissues with lower PPARδ expression (**Figure 1B**).

Inverse relation between the expression of PPARδ and MVD was analyzed using Spearman’s correlation analysis (r = -0.275, P < 0.01, **Figure 1C**). All together, these result imply that PPARδ may inhibit the angiogenesis of CRC.

### Knockdown of PPARδ Stimulates VEGFA Expression in CRC Cells and Promotes HUVEC Angiogenesis

**Figure 2:**
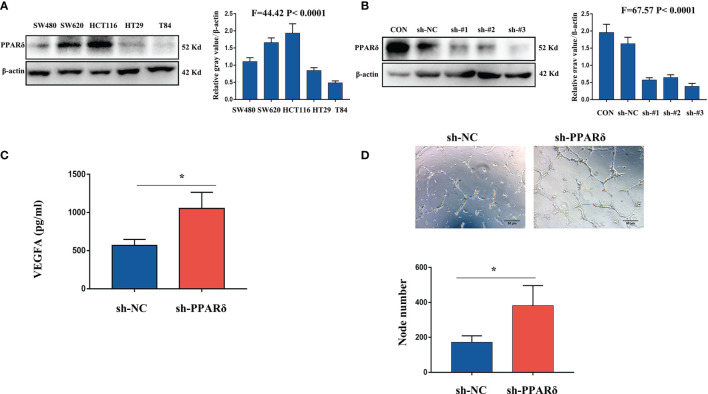
Knockdown of PPARδ promotes HUVEC tube formation through VEGFA. **(A)** Western blot analysis of PPARδ expression in five different CRC cell lines. **(B)** Verification of PPARδ knockdown in HCT116 cells by Western blot analysis. **(C)** VEGFA secretion in conditioned medium of HCT116 cells was examined by ELISA. **(D)** HUVEC tube formation images in the two groups. The number of tubes shows the angiogenesis ability in all groups. Scale bars, 50 μm (magnification, 400×), *P ≤ 0.05.

VEGFA, as a major proangiogenic factor, plays a crucial role in tumor angiogenesis (26). Our previous studies showed that knockdown of PPARδ promoted VEGFA expression in CRC cells *in vivo* (27). We first used lentiviral transfection to establish PPARδ-knockdown HCT116 cells to explore the effect of PPARδ on the secretion of VEGFA in CRC cells. Initially, we measured PPARδ expression in 6 different CRC cell lines. As presented in **Figure 2A**, PPARδ was particularly high in HCT116 cells; hence, PPARδ-shRNA was transduced in HCT116 cells to knock down PPARδ, while a negative control virus was transduced in control cells. Western blotting was used to assessed the knockdown efficiency of all the stably transfected cells. As presented in **Figure 2B**, the strongest efficiency of knockdown was showed in shRNA#3 and it was used for the next study. As VEGFA is a secreted protein, we evaluated VEGFA levels in HCT116 cell-conditioned medium by an ELISA kit following the manufacturer’s instructions and found that knockdown of PPARδ caused significantly higher secretion of VEGFA in conditioned medium than those in PPARδ control cell-conditioned medium (**Figure 2C**).

Next, to *in vitro* evaluate the effect of PPARδ on angiogenesis, a tube formation assay was developed with HUVECs. First, tumor cell-conditioned medium was accumulated from HCT116 cells and mixed it to the culture medium of HUVECs. Compared with in the presence of control cell-conditioned medium, more capillary-like structures were established in the presence of conditioned medium from the PPARδ-knockdown group (**Figure 2D**).

These results revealed that knockdown of PPARδ may promote tube formation in HUVECs through a mechanism that promotes the secretion of VEGFA.

### Knockdown of PPARδ Promoted *In Vivo* Tumor Angiogenesis

We applied the HCT116-PPARδ-knockdown and HCT116-NC cells to establish a nude mice subcutaneous xenograft tumor model. Four- to six-week-old BALB/c nude mice received subcutaneous implantations of HCT116 cells in the right flank. We detected the tumor formation and measured the tumor weight in the two groups.

**Figure 3:**
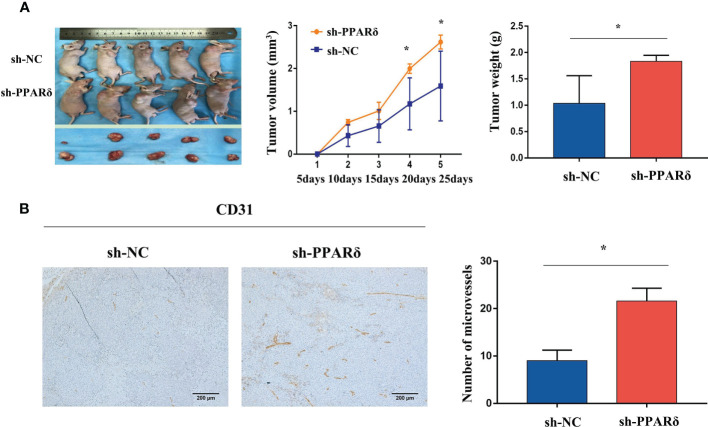
Knockdown of PPARδ promotes nude mice tumor growth and angiogenesis. **(A)** Representative images of tumor-bearing mice and tumor masses. Tumor growth curves were generated. Xenografts’ volumes were measured every 5 days in a 25-day period. **(B)** Representative IHC staining images of CD31 in subcutaneous tumors of nude mice. Scale bars, 200 μm (magnification, 100×). Average tumor weight and microvessels’ number in each group. Data represent the mean ± SD, *P < 0.05.

As expected, the tumor size was increased in PPARδ-knockdown mice when compared with that in control mice(**Figure 3A**). The average tumor volume was significantly higher in PPARδ-knockdown mice than that in HCT116-NC mice (**Figure 3A**). This was consistent with our previous observations in a nude mice xenograft tumor model (27).

Next, to identify the effect of PPARδ on tumor angiogenesis, the tumor sections were stained for CD31, the microvessel marker. The results showed that vessels stained by CD31were more plentiful in the HCT116-PPARδ knockdown groups than in the HCT116-NC group (P < 0.01, **Figure 3B**).

### Knockdown of PPARδ Promoted *In Vivo* Angiogenesis in Matrigel Plugs

**Figure 4:**
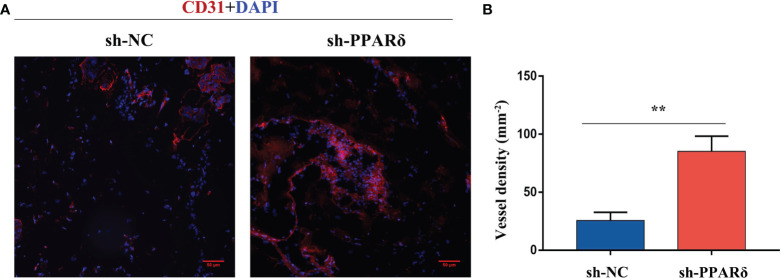
Knockdown of PPARδ enhances angiogenesis as evaluated by a Matrigel plug assay *in vivo*. **(A)** Representative images of CD31 immunofluorescence staining. Cells stained with both DAPI- and CD31 represent endothelial cells. Scale bar, 50 µm. **(B)** Statistical summary of vessel density analysis. The density of CD31-positive cells in the PPARδ-knockdown group was significantly increased compared to the control group (k; mm ^2^; **P < 0.01; Student’s t-test).

To further verify the *in vivo* effects of PPARδ on angiogenesis, an Matrigel plug assay was performed to examine the newly formed vasculature in the transplanted gel plugs (**Supplementary Figures 1**, **2**). HCT116-PPARδ-knockdown or HCT116-NC cells in Matrigel were subcutaneously injected into nude mice. The plugs were harvested 7 days later and assayed for immunofluorescence staining and capillary formation. Staining for CD31 showed that vessel density was higher in PPARδ-knockdown HCT116 cell tumors than in control tumors (**Figure 4A, B**). These results advocated the inhibitory effect of PPARδ on angiogenesis *in vivo*.

### Identification of ERO1A as a Target Gene of PPARδ in the Regulation of VEGFA Secretion by Mass Spectrometry Analysis and ChIP-Seq

**Figure 5:**
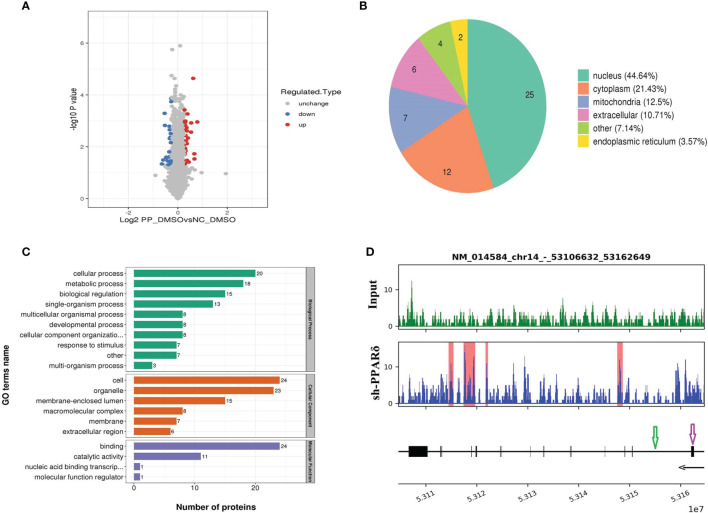
Identification of ERO1A as a direct target gene by mass spectrometry analysis and ChIP-Seq. **(A)** Volcano plot showing differentially expressed proteins between the knockdown and control groups. The upregulated and downregulated proteins (P < 0.05 for both) are shown in red and blue, respectively. Gray represents no significant change in the expression level. **(B)** Differentially expressed proteins’ subcellular localization. **(C)** Functional categorization by Gene Ontology (GO) analysis of the differentially expressed proteins between knockdown and control cells. The GO terms among the three main categories of GO classification (biological process, cellular component and molecular function) are revealed. The x axis represents the number of proteins in a particular GO term within the main category. **(D)** ChIP analysis shows that PPARδ as a transcription factor binds to the intron sites of ERO1A (Gene ID number: 014584) which are highlighted with the red rectangle in the lower panel. The tracks below the lower panel are for the annotations of the chromosomal region of ERO1A. Exon is indicated in purple arrow while Intro in green arrow. The black arrow presents the Transcriptional orientation. Numbers below the black arrow denote chromosomal positions in ERO1A. The binding sites are located between chr14_53106632_53162649.

To investigate the molecular mechanisms underlying the angiogenic effect of PPARδ, we seeked for the PPARδ downstream target gene with LC-MS/MS to identify differentially expressed proteins reacting to PPARδ knockdown. 56 proteins were found differentially expressed(fold change ≥ 1.2 and p-value ≤ 0.05) between the control and PPARδ-knockdown HCT116 cells (30 upregulated and 26 downregulated). These proteins are described in detail in **Figure 3C**. The changes between the two groups was evaluated using K-means clustering heatmaps and a volcano plot, as shown in **Figure 5A, B**.

GO analysis was used to investigate the functional significance of the 56 altered proteins (**Figure 5C**). Among the differentially expressed proteins, heparan sulfate proteoglycan (HSPG) affects the function of the VEGFA-VEGFR2 axis, mainly activating downstream genes (32), and endoplasmic reticulum oxidoreductase 1 alpha (ERO1A) can promote VEGFA production as a key adaptive response under hypoxia (33). Therefore, ERO1A was selected for further research as it was predicted to be targeted by PPARδ and reported to have an effect on promoting the expression of VEGFA.

Next, we examined the direct interaction between PPARδ and ERO1A by ChIP-seq analyses in PPARδ-knockdown HCT116 cells. As PPARδ is a transcription factor, it can increase and decrease target genes’ transcription *via* binding with peroxisome proliferator response elements (PPREs) of target genes, which can also be influenced by chromatin structure, nucleosome localization, and the expression/presence of corepressors, coactivators, and enzymes (34). The results showed that PPARδ-binding motifs were located within ERO1A introns, suggesting that PPARδ may directly repress ERO1A expression (**Figure 5D**).

Together, these results indicated that ERO1A may be the target gene of PPARδ in the regulation of VEGFA expression in CRC.

The GO terms among the three main categories of GO classification (biological process, cellular component and molecular function) are revealed. The x axis means the number of proteins in a particular GO term within the main category. In addition, ChIP analysis shows PPARδ-binding sequences in the introns of ERO1A.

### Knockdown of PPARδ Promotes VEGFA Expression *via* ERO1A

**Figure 6:**
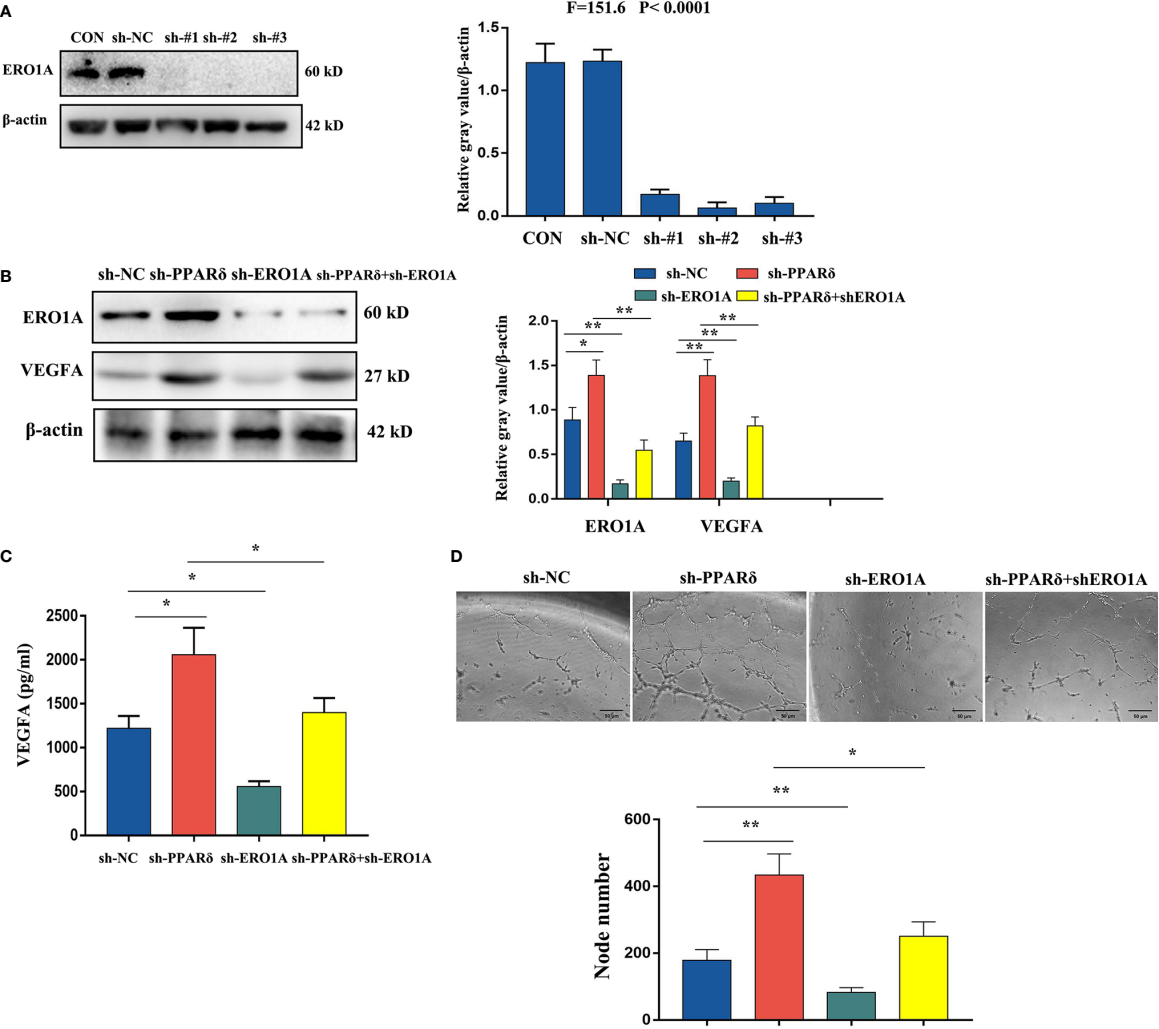
Knockdown of PPARδ promotes VEGFA expression *via* ERO1A. **(A)** Verification of PPARδ and ERO1A codepletion as measured by Western blot analysis. **(B)** The expression of ERO1A and VEGFA in CRC cells as detected by Western blot analysis. **(C)** VEGFA secretion in conditioned medium of HCT116 cells overexpressing both PPARδ and ERO1A as detected by ELISA. **(D)** HUVEC tube formation images in the four groups. The number of tubes shows the angiogenesis ability in all groups. Scale bars, 50 μm (magnification, 400×). The results represent the means ± SD from three separate experiments, *P < 0.05, **P < 0.01.

To confirm whether ERO1A involved the effect of PPARδ on the expression of VEGFA in CRC cells, HCT116 cells with knockdown of both ERO1A and PPARδ through lentiviral transfection were established. The knockdown of both genes was validated by Western blot analyses (**Figure 6A**). Then, WB was used to explore the effects of PPARδ knockdown on ERO1A and VEGFA expression. As shown in **Figure 6B**, ERO1A expression level was increased in the PPARδ-knockdown HCT116 cells compared with the negative control cells. Moreover, knockdown of PPARδ resulted in a rise in the expression levels of VEGFA, (**Figure 6B**). In addition, co-depletion of both PPARδ and ERO1A enhanced the levels of VEGFA compared to depletion of ERO1A alone (**Figure 6B**). These results suggested that the effects of PPARδ on the expression levels of VEGFA depended on ERO1A.

Next, we examined VEGFA levels by ELISA in conditioned medium after 72 h of cell culture. The supernatants of knockdown cells were harvested, centrifuged, and assessed with a human VEGFA ELISA kit. As shown in **Figure 6C**, depletion of ERO1A significantly decreased the secretion of VEGFA in HCT116 cells, while knockdown of PPARδ significantly enhanced the expression of VEGFA. Knockdown of both PPARδ and ERO1A restored the expression of VEGFA close to that in control cells, when compared with knockdown of PPARδ alone. Meanwhile, to determine the changes of angiogenesis *in vitro*, a tube formation assay was developed with HUVECs. After accumulation of tumor cell-conditioned medium from HCT116 cells with knockdown of ERO1A, PPARδ and the both genes, the tumor cell-conditioned medium was mixed to that of HUVECs. Compared with in the presence of control cell-conditioned medium, less capillary-like structures was established in the presence of conditioned medium from the ERO1A-knockdown group while more capillary-like structures were established in that from the PPARδ-knockdown group and subsequently from the knockdown of both ERO1A and PPARδ group(**Figure 6D**). Altogether, these results indicated that PPARδ regulated the secretion of VEGFA in HCT116 cells by targeting ERO1A.

## Discussion

Our recent study implicated PPARδ as a player in the angiogenesis of CRC. However, its exact effect and underlying mechanisms remain unidentified.in this study we demonstrated a significant inverse correlation with MVD in human CRC samples. We identified that knockdown of PPARδ promoted CRC angiogenesis both *in vitro* and *in vivo*. Moreover, mechanistic studies showed that knockdown of PPARδ induced angiogenesis by upregulating VEGFA *via* ERO1A in CRC cells. All together, these findings indicate that PPARδ plays an inhibitory role in CRC angiogenesis, which is one of the mechanisms of suppressing the development of CRC. These results are consistent with those of our earlier reports which also show that PPARδ suppresses CRC carcinogenesis. In accordance with our findings, previous studies have shown that PPARδ inhibited vascular smooth muscle cell proliferation and migration and that PPARδ suppresses angiogenesis in a VEGFR2-dependent manner in human endothelial cells (35, 36). To our knowledge, this is the first study to demonstrate the inhibitory role of PPARδ in CRC angiogenesis.

In contrast with its antiangiogenic effects in CRC, PPARδ has been reported to exert a proangiogenic role in many other tumors. Lung carcinoma was impaired in PPARδ^-/-^ mice, which showed diminished blood flow and an abundance of hyperplastic microvascular structures (37). PPARδ activation can stimulate the expression of VEGFA in breast cancer and prostate cancer (38). This implies that PPARδ may play distinct or even opposite roles depending on the tumor type and environmental context. Similarly, many genes have been reported with context-dependent roles in cancers to either promote or inhibit tumorigenesis. Tumor suppressor candidate 3 (TUSC3) inhibited tumorigenesis in ovarian cancer, prostate cancer, glioblastoma and pancreatic cancer but enhanced cancer progression in head and neck cancer and CRC (39). Toll-like receptors (including TLC1, 2, 3, 4, 5, 7, and 9) have been reported to have both antitumor and protumor effects (40). NOTCH signaling can act as a tumor suppressor or an oncogene in glioma (41). It is difficult to explain such discrepancy; one possible explanation could be the intratumoral heterogeneity (42). It was hypothesized that PPARδ has a dual regulatory role in the angiogenesis of different carcinomas. Given this, further researches are still needed to unravel the effect of PPARδ on tumor angiogenesis.

Further, we performed proteomics to explore the antiangiogenic mechanism of PPARδ in human CRC cells. We found that among the 56 differentially expressed proteins identified in PPARδ-knockdown HCT116 cells, ERO1A and HSPG are related to VEGFA-mediated angiogenesis based on GO and KEGG enrichment analyses. Next, ChIP-seq analyses revealed that PPARδ protein colocalized with ERO1A but not with HSPG *via* protein-chromatin interactions. On the basis of these findings, we speculated that ERO1A could be a target. ERO1A is an oxidase that is contained in endoplasmic reticulum and has an effect on the construction of disulfide bonds in cell-surface and secreted proteins (43). It has been reported as an oncogene in CRC (44) and identified as a poor prognostic factor in several cancers (45–47). Previous studies have stated that tumor angiogenesis was promoted by ERO1A *via* regulating the expression of VEGFA (32, 47, 48). To verify the speculation, we investigated the impact of the coregulation of PPARδ and ERO1A on the expression of VEGFA in HCT116 cells. As predicted, depletion of both PPARδ and ERO1A reversed the effect on VEGFA expression in HCT116 cells mediated by PPARδ depletion alone, suggesting that ERO1A might be involved in PPARδ-regulated expression of VEGFA. Thus, we concluded that the regulatory effect of PPARδ on VEGFA expression was achieved through regulation of ERO1A.

Expression of the key pro-angiogenic factor VEGFA is regulated by hypoxia, growth factors and cytokines, and besides these, mang oncogenes and tumor suppressor genes have been implicated in the regulation of VEGF expression (49–51). The possible regulatory mechanisms of VEGFA in colorectal cancer is shown in the **Supplementary Table 2**. Recognition of the various regulators of VEGFA has developed therapeutic strategies for combination therapy with anti-VEGFA agents to overcome the primary or acquired resistance to antiangiogenic drugs. Anyhow, the frequency of objective responses in patients treated with anti-VEGFA agents alone is modest (8–10). Therefore, our results provide a rational for the combination therapy of PPARδ enhancers with bevacizumab in mCRC to achieve the higher frequency of tumor regressions and optimal clinical benefit.

The main limitation of this study is that the endothelial area was quantified with one marker: CD31. Capillaries should be identified by more than one way to validate our results in further investigations.

In conclusion, our study showed that knockdown of PPARδ could promote angiogenesis through interaction with ERO1A, subsequently increasing the expression of VEGFA. These discoveries support the notion that activation of the PPARδ/ERO1A signaling pathway might be a novel and potential target for antiangiogenic therapy in mCRC.

## Data Availability Statement

The original contributions presented in the study are included in the article/**Supplementary Material**. Further inquiries can be directed to the corresponding authors.

## Ethics Statement

The studies involving human participants were reviewed and approved by Biomedical Ethics Committee of West China Hospital. The patients/participants provided their written informed consent to participate in this study. The animal study was reviewed and approved by Biomedical Ethics Committee of West China Hospital.

## Author Contributions

CF, LY, and ZZ performed study concept and design. WL, DH, JZ, and ZM performed development of methodology and writing, review and revision of the paper. WL and DH provided acquisition, analysis and interpretation of data, and statistical analysis. KC, Zl, and YL provided technical and material support. All authors contributed to the article and approved the submitted version.

## Funding

This study was supported by National Natural Science Foundation of China (grant numbers 81472304), National Key Research and Development Program of China [grant numbers 2016YFC0906000 (2016YFC0906003)], Sichuan Science and Technology Program [grant numbers 2017JY0020], 1.4.5 project for disciplines of excellence, West China Hospital, Sichuan University, and Post-Doctor Research Project, West China Hospital, Sichuan University (2020HXBH078).

## Conflict of Interest

The authors declare that the research was conducted in the absence of any commercial or financial relationships that could be construed as a potential conflict of interest.

## Publisher’s Note

All claims expressed in this article are solely those of the authors and do not necessarily represent those of their affiliated organizations, or those of the publisher, the editors and the reviewers. Any product that may be evaluated in this article, or claim that may be made by its manufacturer, is not guaranteed or endorsed by the publisher.
