# Supplementary figures and images for: Knockdown of PPARδ Induces VEGFA-Mediated Angiogenesis via Interaction With ERO1A in Human Colorectal Cancer
Source: Front Oncol. 2021 Oct 12;11:713892. doi: 10.3389/fonc.2021.713892 (PMC8546184; doi:10.3389/fonc.2021.713892)

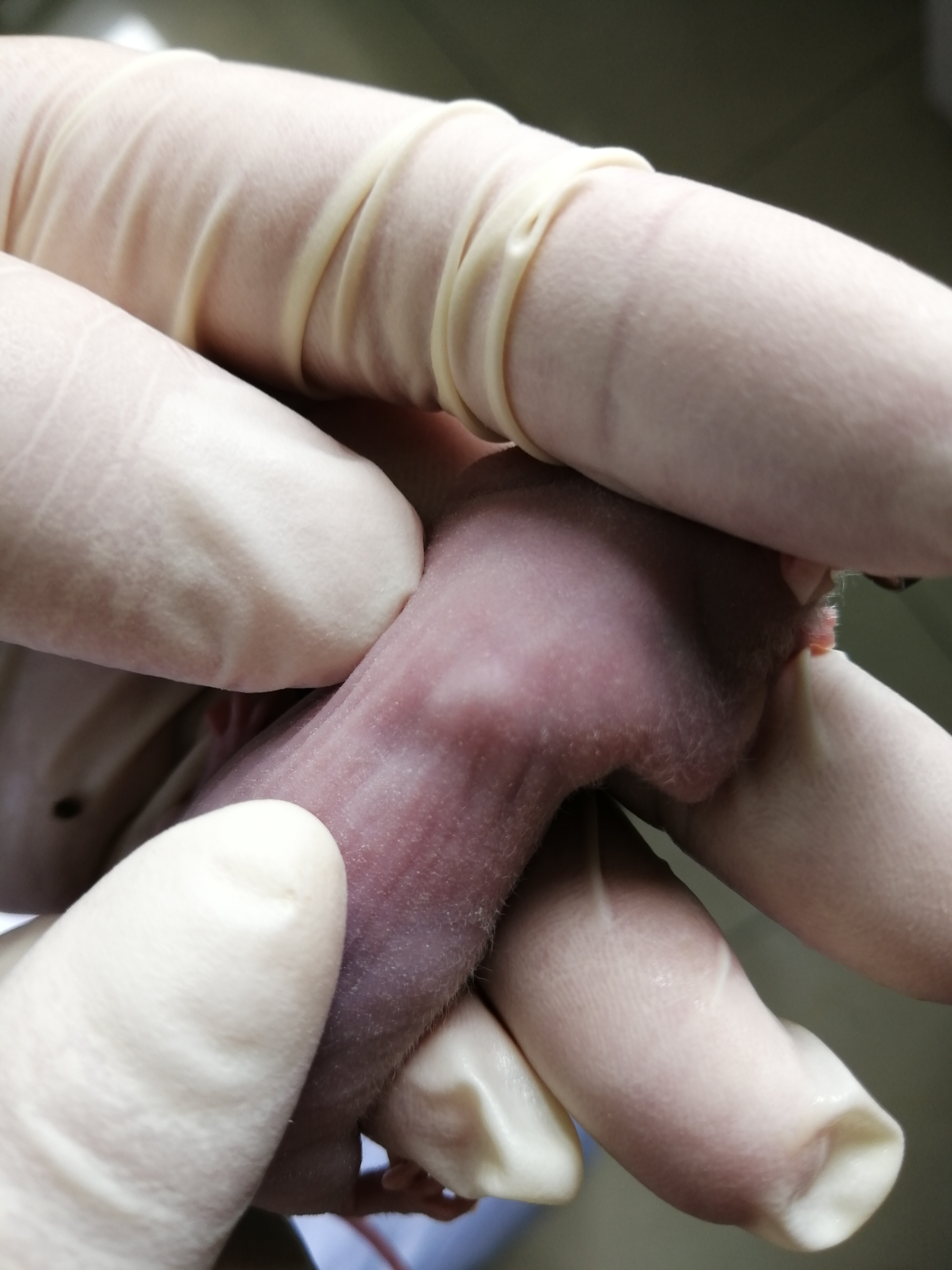

Supplement: Supplementary file 1 [file Image_1.jpeg]

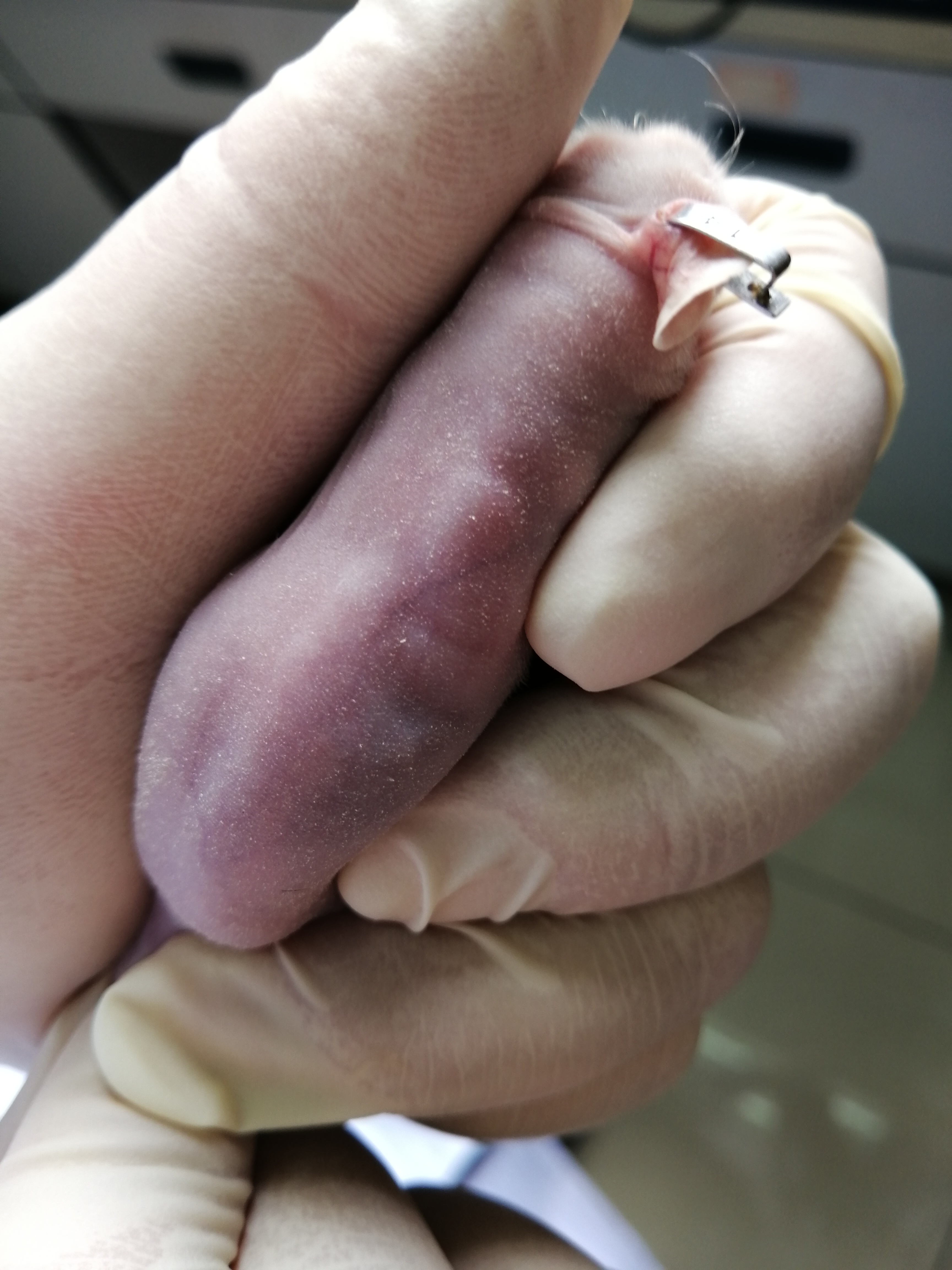

Supplement: Supplementary file 2 [file Image_2.jpeg]
