# Supplementary material for: Knockdown of PPARδ Induces VEGFA-Mediated Angiogenesis via Interaction With ERO1A in Human Colorectal Cancer
Source: Front Oncol. 2021 Oct 12;11:713892. doi: 10.3389/fonc.2021.713892 (PMC8546184; doi:10.3389/fonc.2021.713892)
Supplement: Supplementary file 3 [file Table_1.doc]

Supplementary Table 1. Clinicopathological features of the colorectal cancer cases.

| Variables | Case (n = 120) | Intensity of tumor budding  (Mean ± SD) |
| --- | --- | --- |
| Age |  |  |
| < 65 | 49 | / |
| > 65 | 71 | / |
| Gender |  | / |
| male | 54 | / |
| female | 66 | / |
| Location3 |  | / |
| Left sides | 96 | / |
| Right sides | 24 | / |
| Size (cm) |  | / |
| < 5 | 45 | / |
| > 5 | 75 | / |
| Differentiation |  | / |
| Good+moderate | 63 | / |
| Poor | 57 | / |
| TNM stages |  | / |
| I + II | 62 | / |
| III + IV | 58 | / |
| I | 9 | 1.7±2.1 |
| II | 53 | 4.9±4.2 |
| III | 54 | 7.9±5.4 |
| IV | 4 | 9.3±6.3 |
