# Supplementary material for: Knockdown of PPARδ Induces VEGFA-Mediated Angiogenesis via Interaction With ERO1A in Human Colorectal Cancer
Source: Front Oncol. 2021 Oct 12;11:713892. doi: 10.3389/fonc.2021.713892 (PMC8546184; doi:10.3389/fonc.2021.713892)
Supplement: Supplementary file 4 [file Table_2.doc]

Supplementary Table 2. The most relevant data regarding the regulation of VEGFA in colorectal cancer

| Oncogenes/Tumor Suppressor Genes | Activated Signaling Pathways |
| --- | --- |
| B7-H3 | NF-κB pathway |
| CCL19 | Met/ERK/Elk-1/HIF-1α |
| [miR-148a](https://pubmed.ncbi.nlm.nih.gov/30834693/) | HIF-1α |
| CXCL5 | AKT/NF-κB/FOXD1/ |
| Gab2 | MEK/ERK/c-Myc |
| MicroRNA-181a | SRCIN1/SRC |
| P53 | miR-1249 |
| lncRNA-ZFAS1 | miR-150-5p |
| miR-622 | CXCR4 |
| [MiR-590-5p](https://pubmed.ncbi.nlm.nih.gov/27735951/) | Nuclear factor 90 |
| Akirin2 | IL-6/STAT3/ |
| CD24 | Hsp90/STAT3 |
| CircRNA UBAP2 | miR-199a |
| Histone Deacetylase 1 | HIF1 |
